# Global coal trade induces large CH_4_ emissions

**DOI:** 10.1016/j.isci.2025.112073

**Published:** 2025-02-20

**Authors:** Jinling Guo, Junlian Gao, Kejia Yan, Bo Zhang, He Liu

**Affiliations:** 1 School of Management, China University of Mining and Technology (Beijing), Beijing 100083, China; 2 Faculty of Business Administration, Macau Millennium College, Macau 999078, China; 3 School of Management, China Institute for Studies in Energy Policy, Xiamen University, Fujian 361005, China; 4 The Belt and Road Research Institute, Xiamen University, Fujian 361005, China; 5 National Key Laboratory of Continental Shale Oil, Daqing Oilfield, Daqing 163000, China

**Keywords:** Earth sciences, Environmental science, Environmental monitoring

## Abstract

Coal mining is a major source of global anthropogenic methane (CH_4_) emissions contributing to climate change. The volume of coal traded has reached new heights in recent years, however, existing studies have ignored the impact of CH_4_ emissions induced by coal trade. This study investigates the spatiotemporal evolution of global coal trade-related CH_4_ emissions from 1990 to 2021. Global coal trade-related CH_4_ emissions increased 3.5 times from around 1.4 Tg to 6.1 Tg during this period. Examining past global coal trade activities reveals the extraordinary growth and prominence of the Asia-Pacific region. Australia, Indonesia, and Russia dominated trade-related CH_4_ emissions, accounting for 80.4% in 2021. Hub economies have the potential to drive significant mitigation of trade-related CH_4_ emissions through energy consumption transitions, the deployment of emissions mitigation technologies, and changes in trade structure. Our findings shed light on identifying trade-induced emissions hotspots and managing coal-related CH_4_ to mitigate climate change.

## Highlights

• CH_4_ emissions from coal trade increased from 1.4 Tg in 1990 to 6.1 Tg in 2021
• Russia, Australia, and Indonesia contributed 80.4% of trade-related CH_4_ in 2021
• China and India’s coal imports caused 38.1% of trade-related CH_4_ in 2021
• Trade structure and technical advances have potential to reduce trade-related CH_4_

## Graphical abstract

**Figure:**
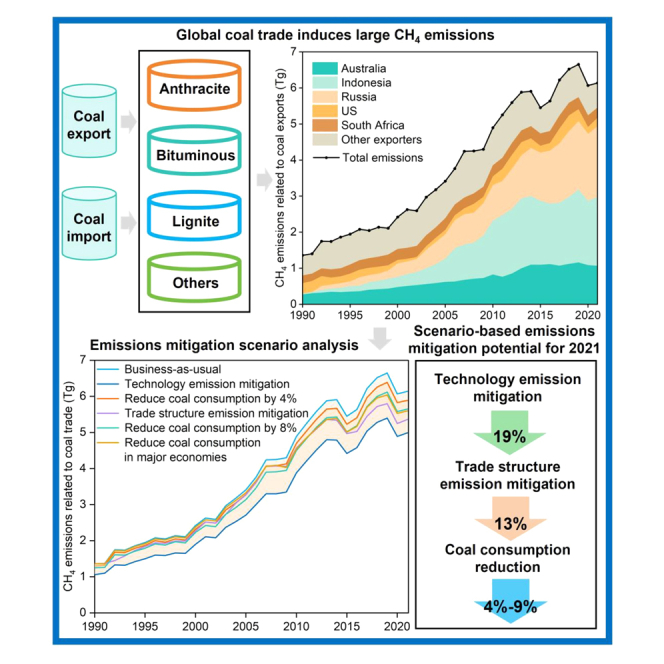

## Introduction

The uneven distribution of energy production and consumption leads to trade in energy internationally,^1^^,^^2^ which provides countries with diversified channels and options for securing energy supplies. With the increasing frequency of cross-border trade flows, a vast coal trade system has gradually emerged. The magnitude of the international coal trade surged by 110% over the period 2000–2021, and this remarkable growth has been driven by the Asia-Pacific region’s hunger for coal.^3^ According to International Energy Agency (IEA) estimates, the coal trade accounted for about 16% of the total coal demand in 2022 and it achieved a new historical record in 2023.^4^ This can be explained by the affordability and availability of coal, which continues to be a key driver of economic growth in many countries, especially in developing countries.^2^^,^^5^

The complex network of the coal trade and the evolution of trade flows have been investigated in previous studies. Song and Wang^6^ and Wang et al.^7^ conducted an in-depth analysis of the spatiotemporal evolution of the global coal resource flow structure. Ekawan et al.^8^ investigated the evolution and future of the hard coal trade in the Asia-Pacific region. Wang et al.^9^ explored the dynamic competitive relationship and intensity through the coal trade globally. The energy trade causes a geographic separation between consumers and the pollution emitted during the production of the traded fuels.^10^^,^^11^ Since the signing of the Paris Agreement, developed countries have embarked on a path of phasing out coal and reducing domestic coal production capacity. However, the high demand for coal in emerging economies has not yet resulted in a significant turning point in global coal trade volume. Stringent climate measures implemented in some countries have reduced coal demand, but these measures may lead to increased emissions elsewhere due to high demand, thereby undermining the effectiveness of mitigation efforts.^12^ Trade flow plays an indispensable role in redistributing global greenhouse gas (GHG) emissions,^11^ with 23–30% of the GHG emissions being embodied in international trade.^13^ Previous studies have confirmed that the global trade in crude oil and natural gas causes energy-related CH_4_ emissions and regional reallocation,^14^^,^^15^ however, the climate impacts due to the global trade in coal remain underexplored.

Since preindustrial times, methane (CH_4_) has been found to be responsible for 0.5-0.6°C of global warming.^16^^,^^17^ The prominent role that CH_4_ emissions play in accelerating climate change has been confirmed by scientific consensus. In 2021, the European Union (EU) and the United States (US) launched the Global Methane Pledge (GMP), pushing the CH_4_ issue to the forefront of global climate governance. The target of the pledge is to reduce global CH_4_ emissions by at least 30% from the 2020 level by 2030,^18^ bringing CH_4_ emissions down to a level consistent with the 1.5°C pathway.^19^ Given the short lifetime of CH_4_, its immediate mitigation will bring relatively direct climate benefits, providing a higher carbon budget for specific temperature control goals.^20^^,^^21^

In response to the pressing issue of CH_4_ emissions, many researchers have identified key emission sources and CH_4_ concentration distribution in the atmosphere through remote sensing^22^^,^^23^^,^^24^ and atmospheric chemical modeling.^25^ According to the Global Methane Budget, annual global CH_4_ emissions from coal mines were estimated to be 42 [29–61] Tg CH_4_ yr^−1^ during 2008–2017, accounting for about one-third of energy-related CH_4_ emissions.^26^ In 2022, satellite detection revealed a large amount of CH_4_ emissions from coal mines in Russia, the US, and other coal-producing regions, with a total of 0.2 Mt and more than 1,500 emissions events.^27^ Satellites have similarly monitored similar CH_4_ emissions hotspots in China. Between 2021 and 2023, satellite data identified 82 major CH_4_ emitters in Shanxi Province, with high annual emissions of up to 1.2 Mt, roughly 4 times that of the world’s largest oil and natural gas CH_4_ emissions hotspots.^28^ Preventing large-scale CH_4_ emissions from coal mines requires more urgent and stringent policy interventions, however, this has largely escaped the notice of policymakers. Globally, mitigation commitments on CH_4_ emissions in the coal industry are not widespread and currently the implementation gap is far too great.^29^ Some participants of the GMP have already taken extensive action in their energy sectors to reduce CH_4_ emissions, but most initiatives in this sector focus primarily on oil and natural gas, with little attention given to coal CH_4_ mitigation. However, addressing CH_4_ emissions from coal mines is more challenging and cannot be overlooked.

Over 80% of the world’s coal resources should remain underground before 2050 in order to meet the global temperature control target of 2°C, or even of 1.5°C.^30^^,^^31^ Although more countries have adopted coal phase-out pledges, this is limited by the particular country’s ability to implement the pledges.^32^ As policymakers seeking to reduce reliance on coal, they may face trade-offs between climate change mitigation, environmental benefits, affordable energy sources, and economic growth.^33^^,^^34^ Given the uncertainty surrounding coal demand in the coming years, deploying effective mitigation measures should be a priority, especially as there are substantial opportunities to significantly reduce emissions in the near term.^35^ Previous studies have examined production-side CH_4_ emissions accounting on different scales using top-down or bottom-up approaches, including regional,^36^^,^^37^ national,^38^^,^^39^^,^^40^^,^^41^ and global inventories.^26^^,^^42^ Existing studies have also identified the main sources of CH_4_ emissions from coal mines, including coal mining (divided into underground and surface mines) and post mining activities. Several studies have conducted in-depth analyses of the evolution trends of CH_4_ emissions from abandoned coal mines.^43^ In addition, the coal sector’s significant potential in reducing CH_4_ emissions^44^^,^^45^ and the co-benefits of emissions mitigation^46^ have been recognized.

A comprehensive review of existing studies into the coal trade and coal mine's CH_4_ emissions mainly focuses on a single perspective, disconnecting the relationship between the coal trade and CH_4_ redistribution, and ignoring the complex dynamics of emissions flows. There are different types of coal within the global coal trade, and considering the variability in coal ranks and emission intensity, this study calls for attention to be paid to CH_4_ emissions in the global coal trade. The study reveals the core economies in the trade market and the characteristics of the spatial and temporal distribution of CH_4_ emissions over the period 1990 to 2021. We aim to shed light on previously unexplored coal-related CH_4_ redistribution from a trade perspective, and to propose policy implications for global and national CH_4_ emission mitigation.

## Results

### Evolution of global coal trade pattern

**Figure 1:**
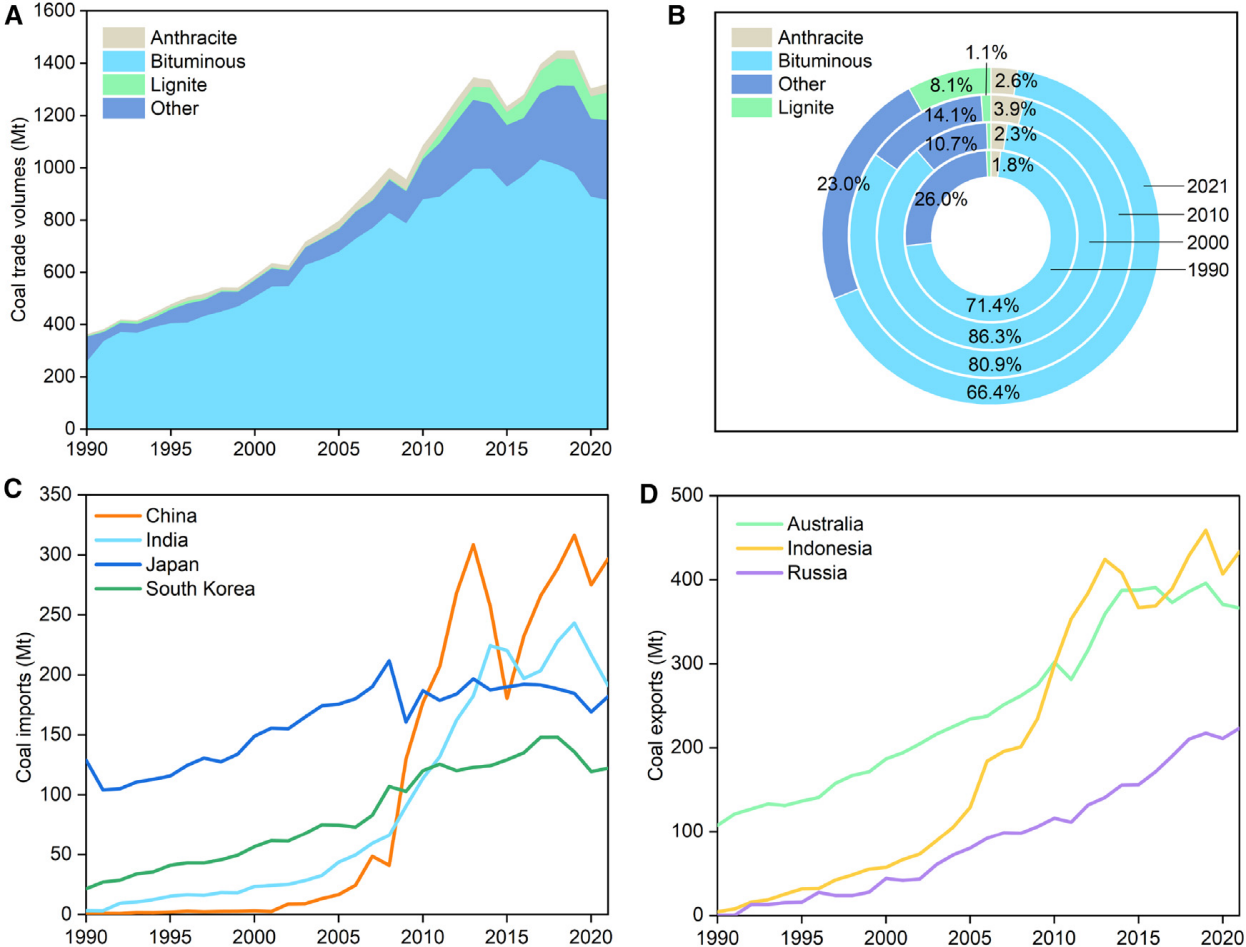
Global coal market trading situation (A) Global coal trade volume from 1990 to 2021. (B) Types of coal traded from 1990 to 2021. (C) Coal imports of major economies from 1990 to 2021. (D) Coal exports of major economies from 1990 to 2021.

The global coal trade volume grew at an average annual rate of about 4.3% from 363.7 Mt in 1990 to 1321.7 Mt in 2021 (Figure 1A). Bituminous coal, favored for its low ash content and high calorific value, is the main type of coal traded internationally, accounting for more than 65% of annual exports, while other types of coal account for about 35% (Figure 1B). Lignite is of low quality and is therefore hardly traded internationally. However, its share in the international market increased from about 1% before 2010 to 8% in 2021. In contrast, the trade share of anthracite has remained stable within a range of about 2%–4% during the period from 2010 to 2021.

**Figure 2:**
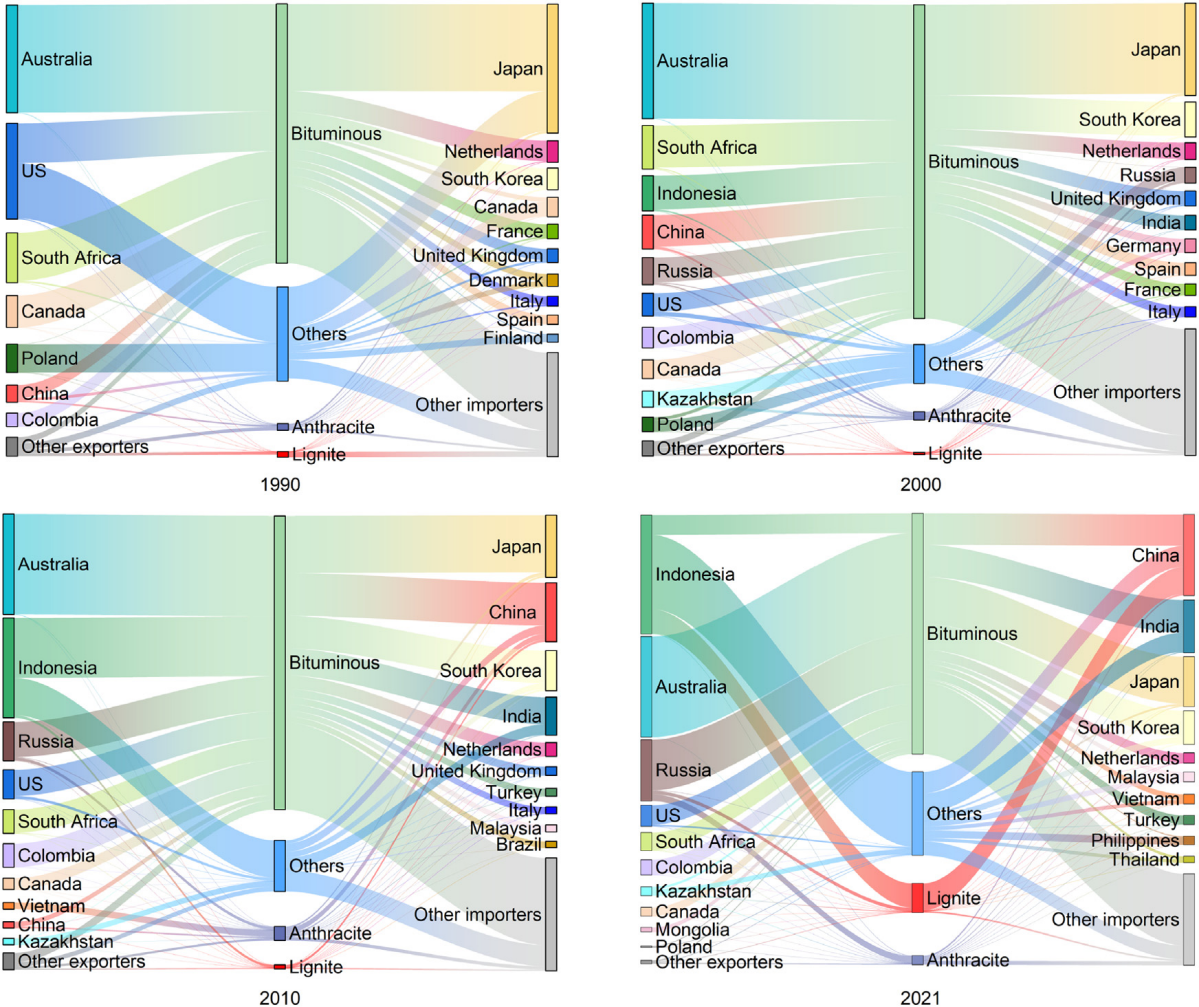
Coal trade flows in the main economies from 1990 to 2021 Note: ‘Others’ represents coal other than anthracite and bituminous, whether or not pulverized but not agglomerated.

The global coal trade flow is determined by the supply and demand allocation of coal resources, that is, the transfer from coal rich areas to demand areas (Figure 2). In 1990, major coal exporters included Australia, the US, and South Africa, accounting for about 69.7% of total global coal trade (Table S1). By 2000, this pattern changed, with Australia, South Africa, Indonesia, and China becoming the dominant exporters, collectively accounting for about 62.9% of total coal trade (Table S1). Japan was the largest importer of coal, while South Korea surpassed the Netherlands in 2000. After 2000, the trend toward global concentration of coal imports and exports has gradually become apparent. The Asia-Pacific region was the most active area in coal trading. Coal importers were concentrated in China, India, Japan, and South Korea, which together accounted for 60% of global coal imports in 2021. Of these, the import share of China and India accounted for about 37% of the world market; Japan’s import share accounted for about 13.8% of the world market; and Korea’s import share remained relatively stable at around 10%. Notably, China’s coal imports exceeded Russian exports in 2021. The export volume of Australia, Indonesia and Russia as a whole showed an increasing trend year by year (Figure 1D, Table S1), and the export volume of the three countries accounted for about 77.4% of world coal exports in 2021 (Table S1). Among them, Indonesia’s coal exports maintained the growth trend, accounting for almost one-third of the world coal export market during 2010–2021.

The coal trade also reflected the movement from low consumption regions to high consumption regions. In 2021, the total coal consumption share of the US, Russia, South Africa, Australia, and Indonesia accounted for only 14.0% of global coal consumption, about one-fifth of the total share of China and India.^3^ In India, coal supported power generation and industrial expansion, and coal demand from the non-power sector tended to outpace domestic coal supply, with the gap being fulfilled by coal imports.^47^ About three-fourths of India’s electricity was coal-based, and imported steam coal plays a vital role in meeting India’s power generation needs.^48^ China showed similar characteristics, with its heavy reliance on coal for power generation and steel production —more than 50% of global production in 2020 for both power generation and steel production— leading to China’s growth as the world’s largest importer of coal for power generation and steelmaking.^49^

### CH_4_ emissions related to global coal trade

**Figure 3:**
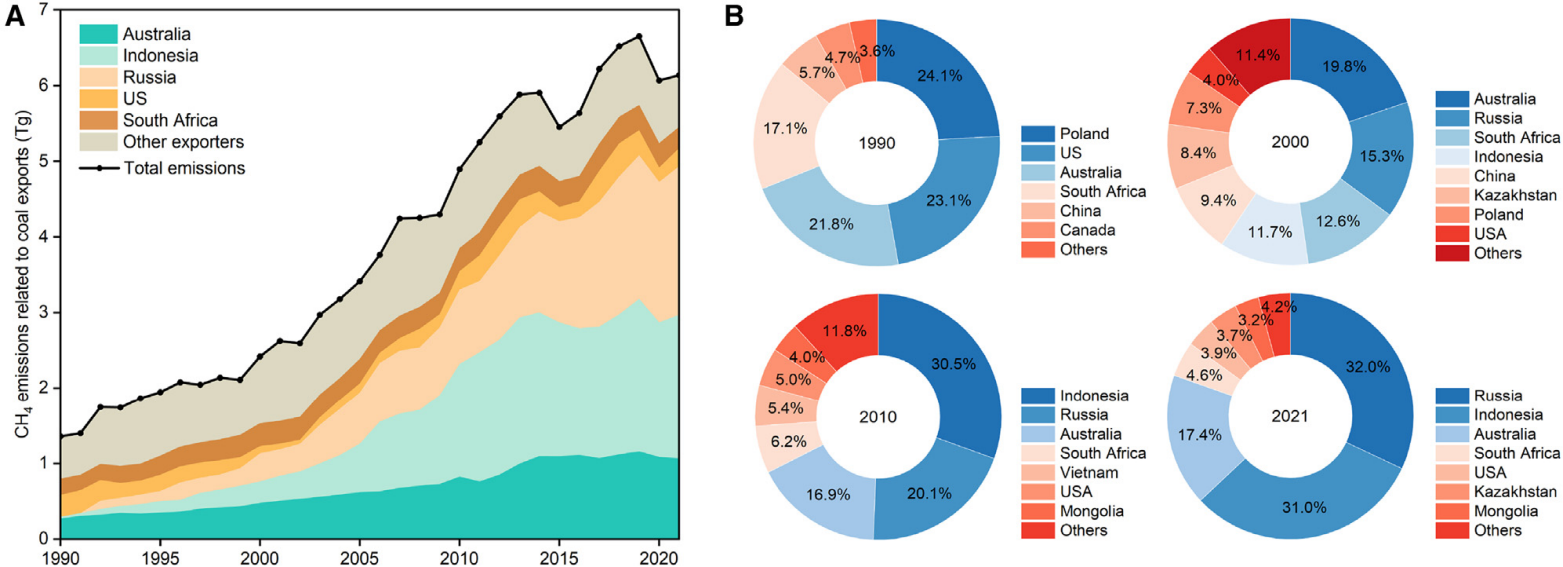
CH_4_ emissions induced by global coal trade from 1990 to 2021 (A) CH_4_ emissions induced by global coal trade. (B) CH_4_ emissions related to coal exports from major economies.

Accurate estimates of CH_4_ emissions associated with the coal trade are critical for future mitigation efforts. The CH_4_ emissions from coal exports experienced a 3.5 times increase, soaring from about 1.4 Tg in 1990 to 6.1 Tg in 2021 (Figure 3A). The CH_4_ emissions from the coal trade in 2021 were found to be 2.2 times higher than those created by the global trade in liquefied natural gas (LNG).^15^ The average annual growth rate of CH_4_ emissions from the coal trade stands at an impressive 5.0%, surpassing the growth rate of coal trading volumes. This discrepancy highlights the significance of CH_4_ transfer induced by trade and warrants close attention. The CH_4_ emissions from the coal trade were directly linked to the volume of trade, and can also be affected by external environmental shocks. For example, after the recovery from the global economic crisis, CH_4_ emissions from the coal trade in 2010 increased by 13.9% over the previous year. Similarly, the impact of the COVID-19 pandemic reduced CH_4_ emissions from the coal trade by 8.8% over the previous year.

Major exporters exhibited distinct variations in CH_4_ emissions (Table S2), influenced by the combined effect of export volume and production emission intensity. As shown in Figure 3B, the contributors of CH_4_ emissions related to the global coal trade gradually became concentrated in Russia, Indonesia, and Australia. By implementing a strategy to expand coal exports, Russia emerged as a major coal exporter, with coal exports accounting for about 16.9% of the total trade and CH_4_ emissions from coal exports accounting for about 32.0% of the total trade-related emissions in 2021. Indonesia was the top global coal exporter in 2021 due to remarkable freight cost advantages and low prices, with the share of CH_4_ emissions related to coal exports ranking behind Russia at 31.0%. Australia experienced the same export growth trajectory due to the higher quality of its coal compared to other exporters’ coal, with CH_4_ emissions accounting for about 17.4% of total trade-related emissions in 2021. Coal exports from these economies have induced more than 70% of trade-related CH_4_ emissions since 2013, which has increased to 80.4% in 2021. This highlights the importance of monitoring and addressing CH_4_ emissions in major coal exporters to effectively reduce trade-related GHG emissions.

**Figure 4:**
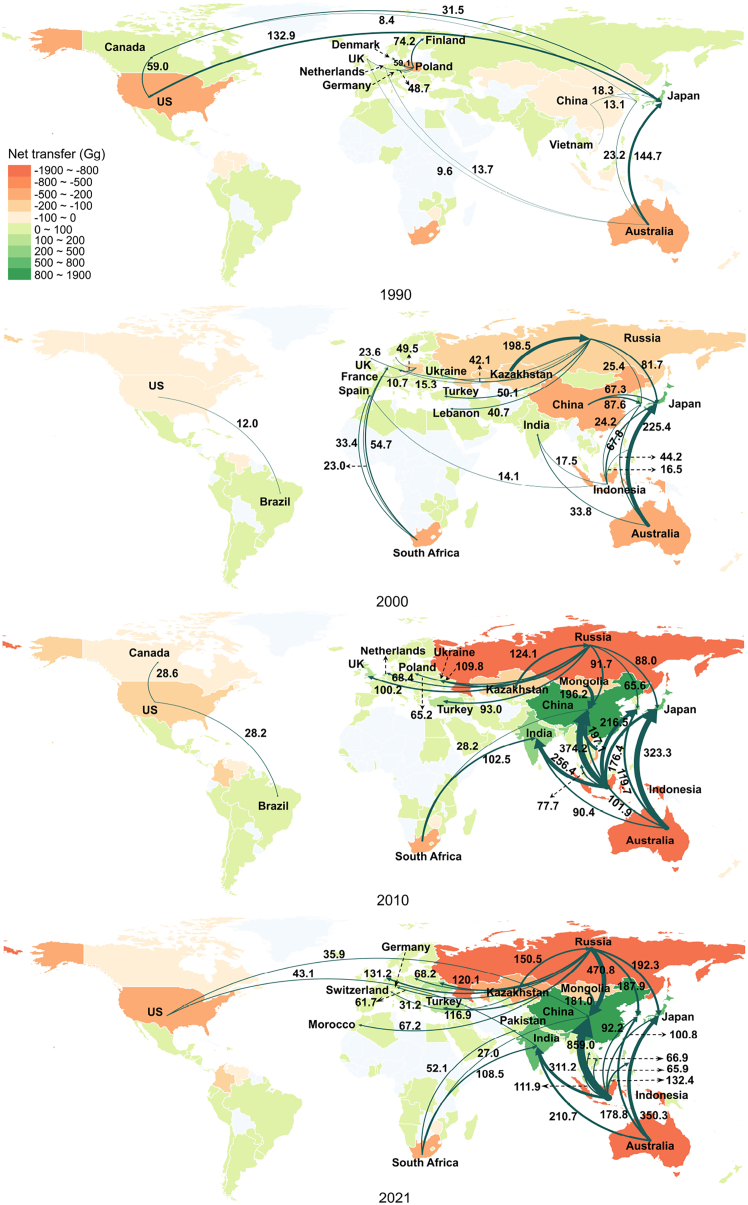
Main CH_4_ emission flows via global coal trade Note: The figure illustrates the main CH_4_ emissions flows related to coal trade in major coal exporters. The trade flows shown in the figure from 1990 to 2021 accounted for about 51%, 52%, 64% and 74% of total CH_4_ emissions for that year, respectively.

The majority of CH_4_ transfers induced by the global coal trade were directed toward the Asia-Pacific region, representing over 70% of total transfers in 2021. Figure 4 shows the spatial transfer of CH_4_ induced by the coal trade in major economies. China, Japan, South Korea, and India were responsible for approximately 57.8% of trade-related CH_4_ emissions induced by coal imports in 2021. China was the largest importer of CH_4_ emissions related to the coal trade, mainly from Russia, Indonesia and Mongolia. China’s ban on Australian coal has found other markets for Australian exporters to transfer more trade-related CH_4_ emissions to India, Japan and South Korea. China’s coal imports induced trade-related CH_4_ emissions amounted to about 1.6 Tg, while the Netherlands, the largest importer in the European region, was responsible for trade-related CH_4_ emissions of about 194.3 Gg. The main reason for this gap was the sustained economic growth in developing countries and the ongoing process of coal phase-out in developed economies, which are gradually fading away from the main coal-consuming markets. The widening gap in coal dependence among economies may pose challenges for future international dialogue, and high coal demanders need to take measures quickly to reduce demand for coal in order to achieve CH_4_ emission mitigation targets.

### CH_4_ emissions related to coal demand in major economies

Both the supply and demand sides of the coal trade play a crucial role in CH_4_ emissions. Emerging economies in Asia are in a period of development, and countries in South and Southeast Asia such as India, Indonesia, and Vietnam are relying on coal to drive economic growth, therefore their demand continues to grow. Economies such as those in Europe, and Canada and the US are on a ‘coal phase-out’ path, benefiting from the growth in renewable energy sources such as photovoltaic wind power and the cleaner nature of natural gas. Although declining, the reduction in demand for coal in these countries has been offset by a significant increase in coal consumption in Asia.^50^ In addition, during the ongoing conflict between Russia and Ukraine, the price of oil and gas in Europe has risen sharply and the importation of natural gas has been restricted. The EU countries restarted coal-fired power generation, and the energy consumption structure inclined toward coal, which, to a certain extent, increased the global coal demand. The coal demand of each country is not only supplied from domestic production, but may also be filled by international trade. Further research on overall CH_4_ emissions from coal demand in major economies is therefore necessary.

**Figure 5:**
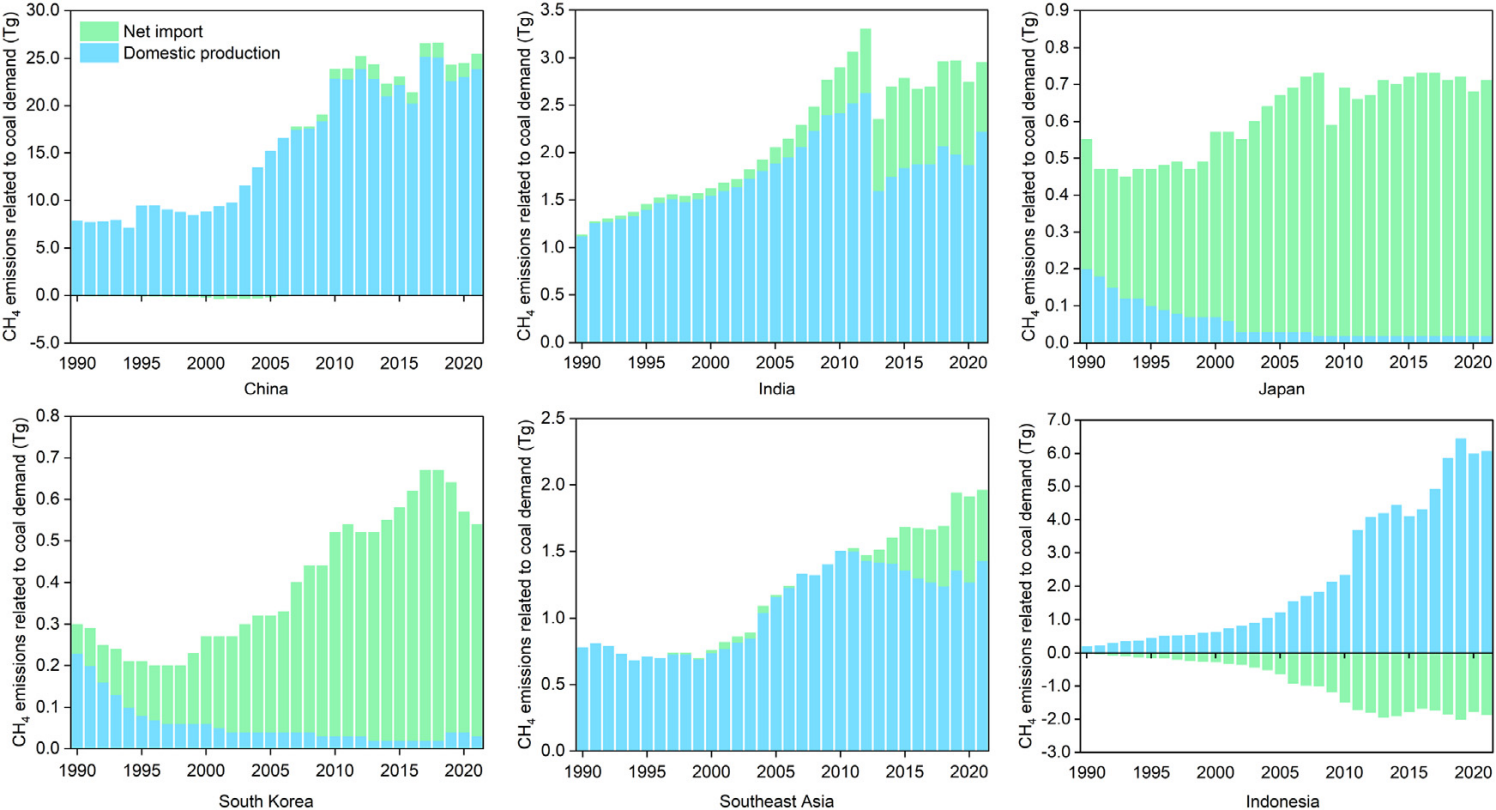
CH_4_ emissions related to coal demand in major economies Note: The countries included in Southeast Asia in the figure are Thailand, Vietnam, Laos, Cambodia, Myanmar, Malaysia and the Philippines.

Figure 5 shows the CH_4_ emissions caused by the coal demand from hub economies in the coal trading system. The coal demand in China, India and Southeast Asian countries was mainly derived from domestic production, supplemented by trade imports. CH_4_ emissions associated with China’s coal demand peaked at 26.6 Tg in 2018. Domestic production, which accounted for more than 90% of the total, was mainly from underground coal mining, and this was the main reason for the higher CH_4_ emissions from coal. Unlike China, India’s CH_4_ emissions from domestic production in 2021 accounted for over 75%, with 96% coming from surface coal mining.^51^ In recent years, due to the high demand for electricity and the development of the steel industry, India’s net coal trade demand has shown an overall growth trend. Unlike emerging economies in Asia, Japan and South Korea’s coal demand was mainly met by trade imports. Japan is totally dependent on the import of fossil fuels, and coal is the least geopolitically risky energy source for Japan, which has long maintained a high level of coal imports. From 2010 to 2021, about 97% of annual CH_4_ emissions related to coal demand in Japan came from imports. South Korea’s coal demand has been on a downward trend in recent years due to a sluggish steel industry and government restrictions on coal-fired power generation dust control.^52^ In 2021, Indonesia’s CH_4_ emissions related to coal demand were approximately 4.2 Tg, surpassing India’s coal demand-related CH_4_ emissions. In addition, the demand for coal in Indonesia and other Southeast Asian countries resulted in about 6.2 Tg of CH_4_ emissions.

Globally, the expansion of coal mining and consumption seems contradictory and inappropriate in the context of the climate crisis. The Asia-Pacific region was a hotspot for coal demand-induced CH_4_ transfers. There was a high degree of variability in coal demand-induced CH_4_ emissions between economies, which was correlated with the geological conditions for coal mining, development of industrial lifelines, and environmental policies. Regardless of whether the coal is produced domestically or imported for trade, underground coal mining generally produces more CH_4_ than surface mining, which needs to be supplemented with certain abatement and recycling technological measures to reduce the amount of CH_4_ released during its operation. Steam coal and coking coal, mainly from underground mining, are the main raw materials for the coal-fired power and steel industries, and coal demand depends on the development of the low-carbon transition of major coal-consuming industries, especially in India and China. On a global scale, only 13% (minimum 10%, maximum 17%) of global CH_4_ emissions were covered by direct CH_4_ mitigation policies.^29^ While energy and environmental issues are intertwined, there is a large gap between the development and implementation of environmental policies, and the failure to fully implement and enforce these policies has become one of the biggest challenges preventing countries from mitigating climate change and reducing pollution.^53^

## Discussion

The Global Warming Potential (GWP) metric, which represents the ability of GHGs to trap heat in the atmosphere in CO_2_ equivalent, was used to estimate the effectiveness of the radiative forcing caused by CH_4_.^54^^,^^55^ According to the IPCC, the GWP of fossil fuel-related CH_4_ is 29.8 times greater than that of the same weight of CO_2_ over a 100-year period and 82.5 times greater over a 20-year period.^54^ The CH_4_ emissions induced by the global coal trade in 2021 amounted to 182.9 Mt CO_2_-eq over the 100-year period, which is equal to Spain’s CO_2_ emissions for that entire year.^56^ At the 20-year time period, the CH_4_ emissions changed to 506.4 Mt CO_2_-eq, which is only 12.6 Mt less than Canada’s CO_2_ emissions for that year.^56^ If we consider the total emissions during the study period, these emissions aggregated to a total of 3.6 Gt CO_2_-eq and 10.1 Gt CO_2_-eq based on the respective GWP values over 100-year and 20-year period.

According to data from the IEA, the global CH_4_ emissions from coal mines reached as high as 40 Mt in 2023 (62.5% from underground coal mining), accounting for approximately 11.3% of anthropogenic CH_4_ emissions.^57^ CH_4_ emissions from coal mining harm the climate, and if not used,^58^ represent the waste of a resource that could be used for energy supply. There are significant near-term emission mitigation opportunities based on existing technologies. However, existing policies and regulations are projected to reduce emissions by less than 10% by 2030, while high-level pledges could lead to a 40% reduction in CH_4_ emissions from coal mines by 2030.^57^ The Global Methane Assessment found that targeted measures could reduce emissions from the coal sector by 12–25 Mt/yr.^59^ It is worth noting that among the top ten coal producing countries, China, India, Russia, South Africa and Poland have not committed to the GMP. These countries accounted for about 66.5% of global coal-related CH_4_ emissions in 2022,^60^ so their participation in emissions mitigation actions is crucial. The main emitters of CH_4_ from coal mines, whether it is the country or enterprise producers, need to strengthen their emissions mitigation actions in this field.

Between 1990 and 2021, global CH_4_ emissions from the trade in coal increased by 4.8 Tg. This increase was directly linked to the trading partners’ choice of importing countries and strongly correlated with the soaring volume of the coal trade. Due to differences in geological conditions, mining methods, deployment of mitigation technologies, and mine management levels, there are large differences in production emission intensity between coal exporters, with emission intensity differences of up to 7 times among different exporters. This also means that imports of products from low emission intensity regions will emit less CH_4_ for the same type and quality of the product. The consumption side has a non-negligible impact on CH_4_ emissions in the coal sector. In the short term, coal will remain the main fuel and raw material in power generation, steel and other industrial fields. Despite the impressive momentum to phase out coal power following the Paris Agreement, the world’s operating coal power capacity has grown 11% since 2015, and global coal use and coal capacity reached an all-time high in 2023.^61^ The steel industry is a major consumer of coking coal, and the annual leakage of CH_4_ from coking coal extraction exceeds that from the world’s gas pipelines and LNG facilities combined.^62^

**Figure 6:**
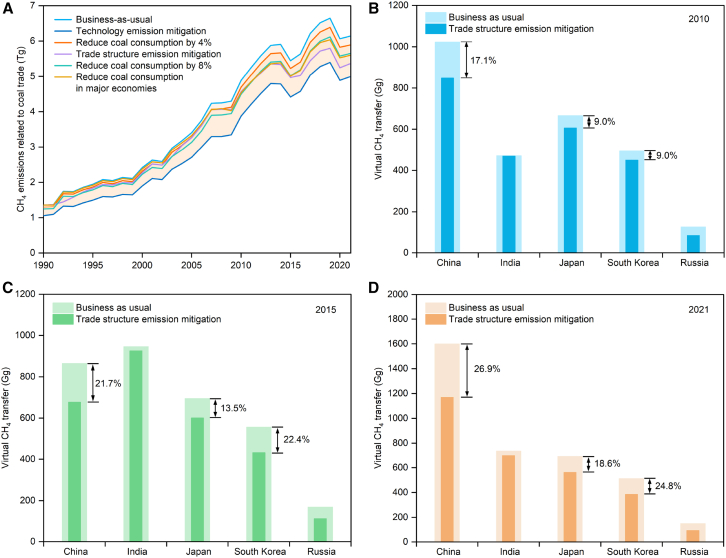
Coal trade-related CH_4_ emission mitigation under different scenarios (A) CH_4_ emission mitigation in coal trade under different measures. (B–D) CH_4_ emission mitigation in major economies from 2010 to 2021 under the trade structure scenario.

Changing the trade structure can contribute to the mitigation of global coal trade-related CH_4_ emissions if importers opt to shift directly to sources with lower emission intensity (Figure 6A). This is a critical way to reverse these flows to reduce the total amount of CH_4_ emissions. Under the specific emissions mitigation scenario related to the trade structure, major economies such as China, India, Japan, and South Korea have considerable CH_4_ emission mitigation benefits from exporters such as Russia and Mongolia (Figures 6A–6D). Global coal trade-related CH_4_ emissions in 2021 would have been reduced by about 12.8% compared to the business-*as*-usual scenario (Table S3), exceeding the total CH_4_ emissions of the Netherlands in that year.^56^ In terms of major economies, China’s coal imports mostly come from Russia, Mongolia, and Kazakhstan due to the China-Australia coal ban in 2021. If China chose to redirect its coal imports from Russia to Australia and replace coal imports from Mongolia and Kazakhstan with Indonesia, this shift would result in about a 26.9% decrease in China’s CH_4_ imports. This change would have a positive impact on CH_4_ emissions from the global coal trade, reducing CH_4_ emissions from coal trading activities by about 7.0%.

Reducing coal production and consumption is the most direct and effective way of mitigating CH_4_ emissions. If the global coal trade had been reduced by 8% in 2021, global coal trade-related CH_4_ emissions would have fallen to 5.7 Tg (Figure 6A). From 1990 to 2021, the coal trade in China and India soared at an average annual growth rate of about 19.5% and 13.9% respectively. If these two countries had reduced their consumption by this proportion each year, they would have reduced CH_4_ emissions by up to 569.5 Gg in 2019. Combined with the reduction of in the coal trade consumption in China, India, Japan, and South Korea, CH_4_ emissions would have been reduced by 9.2% in 2019 compared to the business-as-usual scenario (Table S3), surpassing the total CH_4_ emissions from coal mines in Poland in the current year.^56^ Despite the well understood urgency of the threat of catastrophic climate change, policymakers in newly industrialized countries tend to highlight the importance of coal for industrial development, so coal use is unlikely to decline substantially in the medium term.^50^ Coal seems to be enjoying unbridled growth in India, with the Indian Ministry of Coal forecasting coal demand to reach 1,448 Mt in 2029-2030, an increase of 47.7% from 2021-2022.^51^ In addition, Indonesia, Vietnam, and Turkey have announced plans to significantly expand the use of coal.^63^

Among all mitigation scenarios (Table 1), the decline in exporters’ emission intensity is the most potentially mitigating instrument for global coal trade-related CH_4_ emissions (Figure 6A, Table S3). When exporters reach the production-weighted emission intensity, they can bring 18.7% of the coal trade-related CH_4_ emissions mitigation for 2021 (Table S3), which is equivalent to Japan’s total CH_4_ emissions for that year.^56^ In addition, the EU’s CH_4_ mitigation regulations included the first-ever intensity standard for CH_4_ emissions from energy imports, but the CH_4_ emission intensity standard for imported coal has not yet been explained. The production-weighted emission intensity of the EU coal imports in 2021 was 5.7 kg CH_4_/t, and if coal was imported at this level, CH_4_ imports in the EU region would decrease by 25.3%.

**Table 1:** CH_4_ emission mitigation scenario setting

Scenarios	Scenario description	Emission mitigation target
Business-*as*-usual	CH_4_ emissions scenarios developed in line with original trade trends.	None
Trade structure emission mitigation	Major economies importing coal from Russia shifted to Australia; major economies importing coal from Kazakhstan shifted to Indonesia; major economies importing coal from Mongolia shifted to Indonesia.	Production side: Russia, Mongolia, Kazakhstan; Import side: China, India, Japan, South Korea
Coal consumption reduction	Reduce coal consumption by 4% annually in trade imports.	Global coal importer
	Reduce coal consumption by 8% annually in trade imports.	Global coal importer
	Major economies reduce consumption.	China, India, Japan, South Korea
Technology emission mitigation	The exporters that are higher than the average production emission intensity of the global coal trade, with further mitigation technologies and mine management to achieve the average emission intensity for the given year.	Coal exporters with high emission intensity

Accurate and transparent accounting of CH_4_ emissions from coal trade is linked to the fair distribution of emission mitigation responsibilities among countries, and also directly affects the effectiveness of global climate governance. Coal exporters generate CH_4_ emissions during coal production, but importing countries also indirectly contribute to these emissions through coal demand. Whether following the principles of producer responsibility or consumer responsibility, the aim is to establish a transparent and consistent accounting system which accurately reflects the efforts and effectiveness of all actors in the coal trading chain to reducing trade-related CH_4_ emissions. The GMP has yet to take into account the mitigation of CH_4_ emissions associated with trade activities. Some countries have begun to propose emission mitigation efforts related to oil and gas trade, and have proposed relevant international agreements. For example, the US, South Korea, Australia, the EU, and Japan signed the Joint Statement on Accelerating Methane Mitigation from the LNG Value Chain. Under the framework of the GMP, countries’ emissions mitigation efforts will be assessed on the international stage. A particular focus on trade flows between core economies, such as China and Russia, China and Mongolia, Indonesia and China, Japan and Russia, is essential for reducing global emissions caused by coal trade.

On the production side, coal exporters should clarify their emission mitigation responsibilities and optimize their emission management systems. Governments can introduce mandatory CH_4_ emissions reporting systems for coal producers, requiring them to report their production emissions data comprehensively and systematically. Meanwhile, governments should establish effective assessment systems to ensure that companies take effective measures to reduce emissions. Key mitigation technologies in place within the coal industry and strict mine management systems are vital for a decline in CH_4_ emission intensity from coal mines to occur. In countries without proper mine management, CH_4_ emissions can account for up to 13% of their total GHG emissions along the coal power value chain.^58^ In countries which rely heavily on underground mining, CH_4_ recovery technology is a major way to reduce emissions. For example, China adopted measures to reduce emissions in the coal sector, including installation of drainage systems and preferential treatment of power generation projects, with the aim of increasing the capture, recovery and use of coal mine CH_4_ (CMM)/coalbed CH_4_ (CBM).^29^ CBM became an important supplement to domestic natural gas supply, accounting for about 5% of the domestic gas supply in 2023. In addition, about 60%–80% of the CH_4_ emissions from underground coal mines are emitted as ventilation air CH_4_ (VAM) (typically CH_4_ with the concentration less than 1%).^62^ Strengthening technological innovation to reduce CH_4_ emissions at such concentrations has become an urgent issue which must be addressed.

As an important link in the coal trade chain, importing countries can set stricter import standards and make CH_4_ emission intensity one of the key indicators in procurement standards. This initiative not only directly reduces CH_4_ emissions from coal imports, but also forces coal-producing countries to reduce their emission intensity through market mechanisms. Additionally, major importing economies can strengthen environmental regulation of trade by imposing taxes or reducing tariffs on imported energy to meet CH_4_ emission mitigation targets. While pursuing economic development, countries must seek the optimal balance between coal consumption and CH_4_ emissions control. The existing production processes of industries that rely on coal as the main input still maintain a high demand for coal. In the short term, a significant reduction in coal production on the supply side will affect production activities and economic development, especially in the steel industry, where coking coal as the input, and in the coal-fired power industry, where steam coal as the main fuel. Therefore, the consumption side should actively seek coal alternatives or optimize processes to promote the transformation and upgrading of the coal-dominated energy consumption structure, thereby reducing the pressure on upstream production and supply.

Coal mine CH_4_ mitigation is a global issue, and it is urgent that all countries work together to address this challenge through multi-dimensional and in-depth international cooperation. The international community is called upon to develop a coherent accounting framework for CH_4_ emissions to ensure that countries have uniform standards for calculating trade-related emissions. Data collection and monitoring must be strengthened and combined with real-time monitoring methods to reduce possible errors caused by bottom-up accounting in the coal industry. At the same time, through bilateral or multilateral agreements, the sources of coal production must be scrutinized to ensure transparency of emissions in the coal supply chain. Technological innovation and sharing are important driving forces to promote CH_4_ emission mitigation in coal mines. To help countries with relatively weak emission mitigation capacities improve their technological levels, developed countries should strengthen research and development, and demonstrate and exchange emission mitigation technologies. To translate emission mitigation targets into concrete actions, a cooperative alliance should be established at the national and enterprise levels. The main objective is to establish clear emission mitigation targets and promote emission mitigation actions through multilateral cooperation and joint efforts among enterprises. For example, the Steel/Metcoal Methane Partnership, currently established within the industry, could be extended to major coal-consuming industries and all types of coal to reduce the CH_4_ emission intensity of the coal industry. At the enterprise level, it is recommended that monitoring, reporting, and verification plans be established to evaluate and track the effectiveness of emission mitigation policies. It is also necessary to establish coal traceability, and dynamically track CH_4_ and the environmental, social, and governance (ESG) performance of coal companies.

Compared with the oil and gas industry, global coal-related CH_4_ emission mitigation issues have not received the same level of attention from the international community. This study emphasizes the urgent need for global attention to address CH_4_ emissions associated with the coal trade. Strengthening international cooperation to accelerate the mitigation of CH_4_ emissions is crucial, especially in terms of institutional frameworks and technological innovation. At the same time, to ensure substantial global progress in reducing fossil fuel-related CH_4_ emissions, the wide availability of technical assistance and services is imperative. This support will help countries and industries overcome technical barriers and advance their emissions mitigation targets. We call for the continued monitoring of the CH_4_ footprint in coal trade, as well as broadening the global oil and gas trade to comprehensively review and reduce CH_4_ emissions from these sectors.

### Limitations of the study

The data in this paper was primarily derived from the UN Comtrade database, which provides clear information on trade flows that is of irreplaceable value in understanding trade-related CH_4_ transfer. It is important to note that there is a gap between the UN database and other databases such as the EIA. For example, the US exported about 96.0 Mt of coal in 1990, according to export data from the US Energy Information Administration (EIA) database, but this information was not reflected in the UN Comtrade database. From 1990 to 1992, the volume of coal trade in the UN Comtrade database was about 14% less than that in the EIA database, while the difference in other years was about 7%. This means that we were potentially underestimating the CH_4_ emissions associated with coal trading.

**Figure 7:**
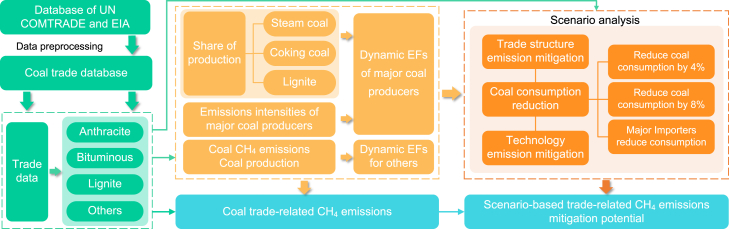
Analysis framework for CH_4_ emissions related to the global coal trade

When accounting for CH_4_ emissions generated by coal trade, the focus was primarily on the key links in the coal production process (Figure 7). We did not account for potential CH_4_ emissions that may occur during transportation, including the release of CH_4_ gas adsorbed by the coal itself and the CH_4_ emissions caused by energy use during transportation. This decision was based on the practical difficulties of obtaining detailed data on coal transportation modes and coal CH_4_ adsorption capacity, as well as the fact that the amount of CH_4_ released from transportation is generally negligible. In addition, the impact of indirectly related coal trading activities on CH_4_ emissions was not fully explored in this study. In particular, the CH_4_ emissions behind the demand for coal indirectly generated through international trade in other commodities such as steel were not included in the scope of the study. Therefore, the results of this study may underestimate the overall CH_4_ emissions of the coal trade to a certain extent. In future research, we aim to broaden the scope to include indirect coal demand and its associated CH_4_ emissions, conducting a comprehensive analysis of the entire life cycle of CH_4_ emissions within the energy supply chain.

## Resource availability

### Lead contact

Further information and requests can be directed to Dr. Bo Zhang (zhangbo@xmu.edu.cn).

### Materials availability

This study did not generate any new physical materials.

### Data and code availability

• All data used in this study is reported in the supplemental information section or obtained from the sources cited in the study.
• The article does not report any new code.
• Any additional information required to reanalyze the data reported in this article is available from the lead contact on request.

## Acknowledgments

This study has been supported by the 10.13039/501100001809National Natural Science Foundation of China (Grant nos. 72088101, 72394405, and 72374175), the 10.13039/501100012166National Key Research and Development Program (No. 2022YFE0127500) and the 10.13039/501100012226Fundamental Research Funds for the Central Universities (Grant nos. 800015A597 and 2022SKGL01).

## Author contributions

Conceptualization, B.Z. and J.G; methodology, J.G. and B.Z.; investigation, J.G., K.Y., and H.L.; writing – original draft, J.G. and J.G.; writing – review and editing, B.Z. and K.Y.; funding acquisition, B.Z. and H.L.; supervision, B.Z. and H.L.

## Declaration of interests

The authors declare no competing interests.

## STAR★Methods

### Key resources table

**Table:** 

REAGENT or RESOURCES	SOURCE	IDENTIFIER
**Deposited data**		

Global coal trade data available in the supplemental information section.	UN COMTRADE database EIA database	Table S1
Coal mine CH_4_ emission factors for traded exporters available in the supplemental information section.	Global Methane Tracker 2023 and EDGAR v8.0	Tables S4 and S5
The share of annual production of steam coal, coking coal and lignite	U.S. EIA	N/A
National level CH_4_ emissions and CO_2_ emissions	UNFCCC	N/A

**Software and algorithms**		

ArcMap 10.8	Commercially Available Software	N/A
Origin 2024	Commercially Available Software	N/A

### Method details

#### CH_4_ emissions estimation

The accounting boundary for coal trade-related CH_4_ emissions in this paper is the CH_4_ emissions associated with upstream coal mining activities induced by coal exports. We estimated the CH_4_ emissions in the global coal trade during 1990–2021 for four major coal categories: anthracite (commodity code HS 270111), bituminous (commodity code HS 270112), lignite (commodity code HS 270210 and 270220) and others (other coals except anthracite and bituminous, commodity code HS 270119). Many classifications are used throughout the world, with the main parameter being the coal rank, supplemented by its intended use, i.e., thermal or metallurgical applications. Coal rank is closely related to coal mine CH_4_ emissions, with higher ranked coal (such as anthracite) having higher CH_4_ content than lower ranked coal (such as lignite). Coal classifications can be divided into steam coal, coking coal, and lignite. Steam coal refers to hard coal used for heat production or steam-raising in power plants and, to a lesser extent, in industry. Coking coal can be used for steelmaking and is a high-quality coal for producing coke. Lignite is used in the power sector mostly in regions near lignite mines due to its low energy content and typically high moisture levels. To enhance the accuracy of emissions, as shown in Figure 7, we introduced the emission intensity of different types of coal for various purposes to modify the dynamic emission factors (EFs) of major coal producers.

In assessing the CH_4_ emission intensity of coal mines, the IEA considers estimates of large emitters detected by satellites and basin-level emissions from coal-producing regions. It provides EFs for different types of coal production in major coal producing countries such as Australia, Indonesia, Russia, and the US. A detailed description of the EFs can be found in the Tables S4 and S5. We use the IPCC Tier 2 method to calculate CH_4_ emissions from the coal trade by multiplying each country’s trading activity data by the country-specific EF. The CH_4_ emissions (*E*) of the global coal trade in year *t* and region *r* are determined by:

(Equation 1) $$E\left(r,t\right)=EF\left(r,t\right)\times AD\left(r,t\right)\times 10^{-6}$$

(Equation 2) $$EF\left(r,t\right)=AD_{s}\left(r,t\right)\times P_{s}\left(r,t\right)+AD_{c}\left(r,t\right)\times P_{c}\left(r,t\right)+AD_{l}\left(r,t\right)\times P_{l}\left(r,t\right)$$

(Equation 3) $$AD\left(r,t\right)=AD_{s}\left(r,t\right)+AD_{c}\left(r,t\right)+AD_{l}\left(r,t\right)$$

(Equation 4) $$E\left(t\right)=\sum_{r}E\left(r,t\right)$$

where *E* refers to CH_4_ emissions in Tg; *EF* refers to CH_4_ EF for coal production in kg CH_4_/t, *AD* refers to coal trade volume in Kt; 10^−6^ refers to unit conversion factor; *P*_*s*_ represents the share of steam coal production in the total coal production, similarly, *c* represents coking coal and *l* represents lignite. The annual production of these three types of coal is obtained from the EIA.^64^ The data from the global coal trade from 1990 to 2021 were derived from the UN COMTRADE and EIA database (Table S1). We selected major coal trade exporters to construct a trade matrix over the period of 1990–2021. In 2021, there were 28 major exporters and 160 importers of global coal trade. The number of coal exporters and their trading partners from 1990 to 2021 was shown in Table S6.

(Equation 5) $$E_{d}\left(r,t\right)=E_{p}\left(r,t\right)+E_{i}\left(r,t\right)-E_{e}\left(r,t\right)$$

where *E*_*d*_ refers to CH_4_ emissions related to coal demand; *E*_*p*_ refers to CH_4_ emissions from domestic coal production; *E*_*i*_ refers to CH_4_ emissions related to coal imports; *E*_*e*_ refers to CH_4_ emissions related to coal exports.

#### Emissions mitigation scenario analysis

We construct scenario assumptions for the growth of trade-related CH_4_ emissions based on the effect of one or more variables, and then determine the degree of influence of each variable based on the difference between the hypothetical results and actual emission trends. The coal production side is the direct physical source of CH_4_ emissions, while the demand on the consumption side indirectly drives the CH_4_ emissions on the production side. The close links between production and consumption constitute a complex supply and demand chain, and trade activities are an important means for coal CH_4_ emissions to spread across borders. Based on the production-consumption perspective, we set up a business-*as*-usual scenario, a technology emission mitigation scenario, a trade structure transformation scenario and a coal consumption reduction scenario, as shown in Table 1.

On the production side, the geological conditions of coal mining vary across countries, but advanced emission mitigation technologies combined with strict mine management can further reduce emission intensity, thus forming a source control of CH_4_ emissions. On the demand side, the global coal trade grew at an average annual rate of 4.3%. If importing countries promoted the energy transition and reduced coal consumption by 4%–8% per year, this would provide important momentum for deeper mitigation of global coal trade-related CH_4_ emissions. In the trade structure transformation, Russia, Mongolia, and Kazakhstan are countries with high production side emission intensity (Table S1), while China, India, Japan, South Korea, and Russia are the main importers. After an adjustment in trade structure, the import sources shift to Australia and Indonesia, which have low emission intensity (Table S1). Rational matching of trade targets is based on the imported coal types of major economies such as China, Japan, India, South Korea and Russia, as well as the major exported coal types of new trade targets in Australia, Indonesia and the US, considering geographical location. A timely adjustment of trade structure and a shift in coal consumption toward low emission intensity production areas can reduce CH_4_ emissions from coal mining achieving real emission mitigation on a global scale.

### Quantification and statistical analysis

Quantitative analysis was conducted using Excel and Origin, and the results were reflected in Figures 1, 2, 3, 5, and 6. Figure 4 utilized ArcMap to display the analysis of trade flow.
