# Supplementary material for: Global coal trade induces large CH4 emissions
Source: iScience. 2025 Feb 20;28(3):112073. doi: 10.1016/j.isci.2025.112073 (PMC11914807; doi:10.1016/j.isci.2025.112073)
Supplement: Document S1. Tables S1–S6 [file mmc1.pdf]

iScience, Volume *28*

Supplemental information

Global coal trade induces large CH_4 emissions

Jinling Guo, Junlian Gao, Kejia Yan, Bo Zhang, and He Liu

Table S1 Trade volume of major global coal exporters (Kt)

Region	1990	1995	2000	2005	2010	2015	2021
Australia	107783.8	136479.8	186745.2	234319.6	301880.9	387849.2	366511.7
Bulgaria	1.0	10.8	0.5	5.7	86.0	72.8	10.6
Canada	32052.8	34104.2	30439.4	28291.2	33410.7	30486.1	31760.5
China	17290.0	28616.5	55046.2	71682.1	18959.4	5197.3	2577.0
Colombia	13505.0	18273.9	33567.5	53662.1	70530.9	72794.2	55600.4
Czechia	2371.9	13912.9	8773.3	6736.2	7553.8	4561.1	1886.8
Germany	5972.4	2411.1	610.0	285.7	1463.7	2519.5	2090.6
Hungary	0.0	1.9	0.1	371.0	13.3	367.2	6.5
India	411.8	651.7	1512.1	1591.9	1144.2	1103.6	882.3
Indonesia	4351.2	31570.5	57152.4	129044.1	298844.4	366970.4	433660.8
Kazakhstan	0.0	20970.0	26238.2	24656.6	18926.7	30356.9	31951.9
Mexico	0.3	0.2	3.9	4.2	109.9	3.1	87.1
Mongolia	0.0	0.2	0.6	2217.8	16726.7	14472.6	16117.6
New Zealand	335.7	1333.8	1551.2	2345.6	2043.8	1403.8	679.3
Poland	28259.0	32334.3	23248.7	19377.0	10663.2	9386.2	6510.6

Table S1 Trade volume of major global coal exporters (Kt) (continued)

Region	1990	1995	2000	2005	2010	2015	2021
Romania	0.0	9.3	0.6	0.7	73.7	388.5	0.2
Russia	0.0	15728.5	43951.0	80212.5	116092.0	156033.4	223406.5
Serbia	0.0	15.7	2.5	129.9	198.1	74.9	197.8
South Africa	49899.9	58748.6	70495.5	75378.7	70754.6	79166.3	65725.6
Spain	3.1	0.6	14.9	57.8	1488.6	1088.8	627.3
Thailand	0.1	0.0	0.1	24.3	21.4	21.2	152.0
Turkey	8.6	0.2	13.8	16.1	42.6	22.4	86.1
Ukraine	0.0	1880.3	2328.9	4980.0	6194.7	564.1	1.0
United Kingdom	925.9	859.5	950.6	578.9	718.5	384.2	1134.1
USA	95981.9	74684.7	36613.9	47769.7	87135.5	67104.8	77299.7
Venezuela	1834.0	3648.8	5185.9	6218.6	1881.1	1115.6	394.3
Vietnam	2639.5	1845.2	3220.7	7584.3	19717.2	1727.2	1812.5
Zimbabwe	66.0	119.8	16.6	62.0	113.3	85.3	504.1

Table S2 CH_4 emissions from coal exporters (Gg)

Region	1990	1995	2000	2005	2010	2015	2021
Australia	272.7	352.2	479.7	620.6	826.3	1097.1	1068.5
Bulgaria	0.0	0.0	0.0	0.0	0.2	0.2	0.0
Canada	51.7	54.2	47.1	43.5	51.7	46.4	54.2
China	71.8	118.8	228.5	297.5	78.7	21.6	10.3
Colombia	25.0	33.8	61.9	98.7	130.7	134.9	104.6
Czechia	6.5	33.6	16.3	12.4	13.4	8.4	3.8
Germany	25.2	9.2	2.0	0.7	3.1	6.2	4.0
Hungary	0.0	0.0	0.0	0.6	0.0	0.6	0.0
India	2.2	3.1	6.7	6.9	5.2	5.0	3.8
Indonesia	21.4	152.3	282.9	642.0	1495.1	1774.6	1903.0
Kazakhstan	0.0	175.1	204.3	185.5	141.5	220.3	229.3
Mexico	0.0	0.0	0.0	0.0	0.6	0.0	0.5
Mongolia	0.0	0.0	0.0	19.1	197.0	166.6	194.9
New Zealand	1.1	4.3	5.1	7.6	6.7	4.6	2.2
Poland	303.7	311.0	175.9	120.2	56.4	48.5	32.4

Table S2 CH_4 emissions from coal exporters (Gg) (continued)

Region	1990	1995	2000	2005	2010	2015	2021
Romania	0.0	0.0	0.0	0.0	0.3	1.3	0.0
Russia	0.0	132.2	371.2	678.6	985.2	1337.9	1963.5
Serbia	0.0	0.1	0.0	0.3	0.5	0.2	0.5
South Africa	215.3	253.2	303.7	324.6	304.9	342.2	283.0
Spain	0.0	0.0	0.1	0.2	6.2	3.3	1.1
Thailand	0.0	0.0	0.0	0.0	0.0	0.0	0.3
Turkey	0.1	0.0	0.1	0.1	0.2	0.1	0.4
Ukraine	0.0	16.7	17.3	43.1	54.7	4.8	0.0
United Kingdom	7.4	5.5	6.0	2.6	2.3	1.6	6.5
USA	290.1	214.9	97.4	123.4	243.6	187.4	239.0
Venezuela	24.4	48.5	68.9	82.7	25.0	14.8	5.2
Vietnam	40.3	26.3	44.9	102.0	265.0	23.3	24.7
Zimbabwe	0.3	0.6	0.1	0.3	0.6	0.4	2.7

Table S3 Coal trade-related CH_4 emission mitigation in different scenarios (Tg)

Scenarios	Trade structure emission mitigation	Technology emission mitigation	Reduce coal consumption by 4%	Reduce coal consumption by 8%	Reduce coal consumption in major economies
1990	0.00	0.29	0.05	0.11	0.00
1991	0.00	0.27	0.05	0.11	0.01
1992	0.30	0.42	0.07	0.14	0.02
1993	0.17	0.42	0.07	0.14	0.02
1994	0.13	0.44	0.07	0.15	0.02
1995	0.11	0.44	0.08	0.16	0.03
1996	0.14	0.48	0.08	0.17	0.03
1997	0.11	0.46	0.08	0.16	0.03
1998	0.15	0.48	0.09	0.17	0.03
1999	0.12	0.46	0.08	0.17	0.03
2000	0.15	0.52	0.10	0.19	0.04
2001	0.11	0.52	0.11	0.21	0.04
2002	0.11	0.51	0.10	0.21	0.05
2003	0.24	0.60	0.12	0.24	0.05
2004	0.14	0.65	0.13	0.25	0.06
2005	0.15	0.70	0.14	0.27	0.09
2006	0.15	0.77	0.15	0.30	0.11
2007	0.18	0.94	0.17	0.34	0.18
2008	0.19	0.95	0.17	0.34	0.16
2009	0.23	0.95	0.17	0.34	0.27
2010	0.32	1.02	0.20	0.39	0.35
2011	0.40	1.05	0.21	0.42	0.41
2012	0.50	1.08	0.22	0.45	0.48
2013	0.52	1.08	0.24	0.47	0.52
2014	0.56	1.11	0.24	0.47	0.52
2015	0.48	1.03	0.22	0.44	0.44
2016	0.61	1.06	0.23	0.45	0.47
2017	0.77	1.18	0.25	0.50	0.52
2018	0.80	1.24	0.26	0.52	0.56
2019	0.85	1.25	0.27	0.53	0.61
2020	0.82	1.18	0.24	0.49	0.54
2021	0.78	1.15	0.25	0.49	0.53

Table S4 Emission factors for global coal trade exporters (kg CH_4 / t)

Region	1990	1995	2000	2005	2010	2015	2021
Australia	2.53	2.58	2.57	2.65	2.74	2.83	2.91
Bulgaria	5.67	4.11	3.64	3.19	2.61	2.59	2.63
Canada	1.61	1.59	1.55	1.54	1.54	1.52	1.71
China	4.15	4.15	4.15	4.15	4.15	4.15	4.01
Colombia	1.85	1.85	1.84	1.84	1.85	1.85	1.88
Czech Republic	2.75	2.41	1.86	1.84	1.78	1.83	1.99
Germany	4.22	3.80	3.24	2.54	2.10	2.47	1.89
Hungary	2.12	1.86	1.92	1.73	1.72	1.71	1.98
India	5.38	4.74	4.43	4.33	4.50	4.55	4.31
Indonesia	4.92	4.82	4.95	4.98	5.00	4.84	4.39
Kazakhstan	11.31	8.35	7.79	7.52	7.48	7.26	7.18
Mexico	7.20	4.23	4.30	4.98	5.00	3.11	5.92
Mongolia	3.67	6.56	2.24	8.63	11.78	11.51	12.09
New Zealand	3.38	3.25	3.26	3.25	3.25	3.29	3.25
Poland	10.75	9.61	7.56	6.21	5.29	5.16	4.97

Table S4 Emission factors for global coal trade exporters (kg CH_4 / t) (continued)

Region	1990	1995	2000	2005	2010	2015	2021
Romania	5.46	4.27	3.90	3.71	3.36	3.41	3.48
Russia	8.41	8.41	8.44	8.46	8.49	8.57	8.79
Serbia	3.82	3.78	2.59	2.59	2.69	2.68	2.68
South Africa	4.31	4.31	4.31	4.31	4.31	4.32	4.31
Spain	5.31	4.84	4.84	4.24	4.13	3.04	1.78
Thailand	1.68	1.67	1.68	1.67	1.69	1.71	1.73
Turkey	6.75	6.08	5.70	5.64	5.18	5.26	5.11
Ukraine	8.04	8.89	7.44	8.66	8.84	8.41	12.11
United Kingdom	8.01	6.38	6.29	4.48	3.16	4.17	5.71
USA	3.02	2.88	2.66	2.59	2.79	2.79	3.09
Venezuela	13.30	13.29	13.29	13.29	13.29	13.29	13.29
Vietnam	15.26	14.24	13.95	13.45	13.44	13.47	13.60
Zimbabwe	4.74	4.75	4.78	4.81	4.86	4.76	5.28

Table S5 Emissions intensities of major coal producers (kg CH_4 / t of coal equivalent)

Region	Steam coal	Coking coal	Lignite
Australia	3.8	5.4	0.4
China	5.2	10.4	0.5
India	5.7	18.8	0.6
Indonesia	7.7	15.7	—
Russia	12	22.1	1.2
South Africa	6	9.3	—
United States	3.3	15.2	0.3

Note: The data is sourced from the Global Methane Tracker 2023.

Table S6 Overview of coal exporters and their trading partners during 1990-2021

Year	Exporters	Importers
1990	24	87
1991	24	88
1992	27	101
1993	24	105
1994	26	112
1995	28	120
1996	28	123
1997	28	118
1998	28	124
1999	28	122
2000	28	140
2001	28	143
2002	28	152
2003	28	151
2004	28	150
2005	28	156
2006	28	140
2007	28	155
2008	28	153
2009	28	160
2010	28	165
2011	28	164
2012	28	162
2013	28	161
2014	28	157
2015	28	157
2016	28	155
2017	28	161
2018	28	156
2019	28	154
2020	28	150
2021	28	160
